# CD8+CD122+PD-1+ Tregs Synergize With Costimulatory Blockade of CD40/CD154, but Not B7/CD28, to Prolong Murine Allograft Survival

**DOI:** 10.3389/fimmu.2019.00306

**Published:** 2019-02-26

**Authors:** Huazhen Liu, Feifei Qiu, Yuanzhong Wang, Qiaohuang Zeng, Cuihua Liu, Yuchao Chen, Chun-Ling Liang, Qunfang Zhang, Ling Han, Zhenhua Dai

**Affiliations:** ^1^Section of Immunology and Joint Immunology Program, Guangdong Provincial Academy of Chinese Medical Sciences, Guangdong Provincial Hospital of Chinese Medicine, Guangzhou, China; ^2^Department of Cancer Biology, Beckman Research Institute of City of Hope, Duarte, CA, United States

**Keywords:** regulatory T cell (Treg), costimulatory blockade, allograft survival, transplantation immunology, PD-1

## Abstract

A transplanted organ is always rejected in the absence of any immunosuppressive treatment due to vigorous alloimmunity. However, continuously global immunosuppression with a conventional immunosuppressant may result in severe side effects, including nephrotoxicity, tumors and infections. Tregs have been widely used to inhibit allograft rejection, especially in animal models. However, it's well accepted that administration of Tregs alone is not satisfactory in immune-competent wild-type animals. Therefore, it's imperative to promote Treg therapies under the cover of other approaches, including costimulatory blockade. In the present study, we demonstrated that administration of *in vitro*-expanded CD8^+^CD122^+^PD-1^+^ Tregs synergized with costimulatory blockade of CD40/CD154, but not B7/CD28, to prolong skin allograft survival in wild-type mice and to reduce cellular infiltration in skin allografts as well. Treg treatment and blockade of CD40/CD154, but not B7/CD28, also exhibited an additive effect on suppression of T cell proliferation *in vitro* and pro-inflammatory cytokine expression in skin allografts. Importantly, blocking B7/CD28, but not CD40/CD154, costimulation decreased the number of transferred CD8^+^CD122^+^PD-1^+^ Tregs and their expression of IL-10 in recipient mice. Furthermore, it's B7/CD28, but not CD40/CD154, costimulatory blockade that dramatically reduced IL-10 production by CD8^+^CD122^+^PD-1^+^ Tregs *in vitro*, suggesting that B7/CD28, but not CD40/CD154, costimulation is critical for their production of IL-10. Indeed, infusion of IL-10-deficient CD8^+^CD122^+^PD-1^+^ Tregs failed to synergize with anti-CD154 Ab treatment to further prolong allograft survival. Our data may explain why blocking B7/CD28 costimulatory pathway does not boost IL-10-dependent Treg suppression of alloimmunity. Thus, these findings could be implicated in clinical organ transplantation.

## Introduction

Organ transplantation provides an ultimate solution for patients with end-stage organ diseases (1, 2). However, transplanted patients usually need continuous immunosuppression with traditional immunosuppressive agents in order to suppress transplant rejection. Although conventional immunosuppressive drugs can inhibit allograft rejection and improve transplantation outcome via different mechanisms (3), continuously global immunosuppression may cause severe side effects, including nephrotoxicity, tumors, and infections. Previous studies have demonstrated that CD8^+^CD122^+^ T cells also are regulatory T cells (Tregs) that control immunological homeostasis, inhibit conventional T cell responsiveness (4–9) and regulate autoimmune responses (10, 11), including experimental autoimmune encephalomyelitis (11–13), Graves' disease (14), and Colitis (9). We recently found that CD8^+^CD122^+^ Tregs were more potent in suppression of allograft rejection than were CD4^+^FoxP3^+^ Tregs (15). Furthermore, we have shown that a PD-1^+^ component within CD8^+^CD122^+^ population is mainly responsible for suppression mediated by CD8^+^CD122^+^ Tregs (16). However, cellular therapies with either CD8^+^CD122^+^ or CD4^+^CD25^+^ Tregs alone usually do not achieve satisfactory outcome in immune-competent wild-type animals. Therefore, it is necessary to further boost the suppressive effects of CD8^+^CD122^+^ PD-1^+^ Tregs under the cover of additional measures, including costimulatory blockade.

In this study, we investigated the joint effects of administration of CD8^+^CD122^+^PD-1^+^ Tregs and conventional costimulatory blockade on allograft rejection and alloimmunity. We found that CD8^+^CD122^+^PD-1^+^ Tregs synergized with costimulatory blockade of CD40/CD154, but not B7/CD28, to prolong skin allograft survival in wild-type mice. Administration of the Tregs and CD154 blockade also exhibited an additive effect on suppression of T cell proliferation *in vitro* and pro-inflammatory cytokine expression in skin allografts. We further demonstrated that costimulatory blockade of B7/CD28, but not CD40/CD154, negatively impacted adoptively transferred Treg expansion *in vivo* and their production of IL-10 *in vitro*. Finally, the additive effects of CD8^+^CD122^+^PD-1^+^ Tregs and the costimulatory blockade on allograft survival are largely dependent on IL-10 expression by the Tregs.

## Materials and Methods

### Animals

C57BL/6 and BALB/c mice (6–8 week-old and male weighing 20 ± 3 g) were purchased from Guangdong Medical Laboratory Animal Center (Guangzhou, Guangdong, China) while IL-10–/– and Thy1.1+ mice in a C57BL/6 background were purchased from the Jackson Laboratory (Bar Harbor, ME). All mice were housed in specific pathogen-free rooms with controlled conditions. All experiments were approved by the Institutional Animal Ethical Committee of Guangdong Provincial Academy of Chinese Medical Sciences.

### Skin Transplantation

Skin donors were 6–8 week-old BALB/c mice while skin recipients were C57BL/6 mice with the similar ages. Full-thickness trunk skin with an approximate size of 10 mm^2^ was transplanted to the dorsal flank area of recipient mice and secured with a bandage of Band-Aid (Johnson Johnson, New Brunswick, NJ). The bandage was removed 8 days after transplantation. Skin allograft rejection was monitored daily and defined as graft necrosis > 90%, as described previously (17).

### Preparation and Purification of CD8^+^CD122^+^PD-1^+^ Tregs

To isolate CD8^+^CD122^+^ cells, splenocytes from C57BL/6 mice were isolated and stained with anti-CD8-PE and anti-CD122-FITC Abs (BD Biosciences). CD8^+^CD122^+^ cells were then purified using FACSAria III cell sorter (BD Biosciences). Cells (4 × 10^5^ cells/well) were then stimulated with irradiated (3,000 rad) and T-cell-depleted BALB/c splenocytes (4 × 10^5^ cells/well) in 96-well plates in complete RPMI-1640 medium (Gibico, USA), containing 10% fetal bovine serum, 100 U/ml penicillin and 100 μg/ml streptomycin, in the presence of IL-2 (5 ng/ml) and IL-15 (10 ng/ml) for 5 days. The cells were stained with anti-CD8-PE, anti-CD122-FITC, and anti-PD-1-APC Abs (all from BD Biosciences) and analyzed via a FACSCalibur. For adoptive transfer experiments, CD8^+^CD122^+^PD-1^+^ Tregs were finally sorted from the cultured cells via FACSAria III cell sorter. The purity of sorted cells was typically >96%.

### Treatment of Mice

C57BL/6 mice were transplanted with BALB/c skin and then treated *i.p* with either CTLA4-Ig or MR1 (Anti-CD154) (Bio-X-Cell, West Lebanon, NH) at 0.25 mg on days 0, 2, 4, 6, and 8 following skin transplantation. Some recipient mice received *i.v*. 2 × 10^6^ CD8^+^CD122^+^PD-1^+^ Tregs upon skin transplantation with or without the treatment with CTLA4-Ig or MR1. Others were treated *i.p* with anti-IL-10 Abs at 0.1 mg also on days 0, 2, 4, 6, and 8 following skin transplantation.

### Histological Analysis

Skin grafts of recipient mice were harvested, fixed with 4% paraformaldehyde for around 24–48 h and then embedded in paraffin. The 3 μm sections of skin tissues were then made and stained with Hematoxylin and Eosin (H&E).

### Treg Suppression of T Cell Proliferation in an MLR *in vitro*

Nylonwood-enriched T cells (4 × 10^5^ cells/well) from C57BL/6 mice, as responders, were cultured with irradiated BALB/c spleen cells (stimulators: 4 × 10^5^ cells/well) in 96-well plates (Corning Costar, NY) in the complete RPMI-1640 medium (10% FCS, 2 mM glutamine, 100 U/ml penicillin, and 100 μg/ml streptomycin). In some groups, CD8^+^CD122^+^PD-1^+^ Tregs (2 × 10^5^ cells/well) from C57BL/6 mice were also added to the culture as suppressors in the absence or presence of MR1 or CTLA4-Ig (2.5 μg/ml). Five days later, cells were harvested and analyzed using a Scintillation counter (PerkinElmer, Meriden, CT). Cells were pulsed with [^3^H]-Thymidine for the last 8 h before harvesting.

### Flow Cytometric Analysis

To track transferred Thy1.1^+^ Tregs in recipient mice, draining lymph node (LN) and spleen cells were harvested, stained with anti-CD8-PE and anti-Thy1.1-FITC antibodies (BD Biosciences, USA) and analyzed to quantify CD8^+^Thy1.1^+^ Tregs using a flow cytometer (FACSCalibur, BD Biosciences).

### Measurement of IL-10 via ELISA

CD8^+^CD122^+^PD-1^+^ Tregs (2 × 10^5^ cells/well) were cultured in 96-well plates in complete RPMI-1640 medium and stimulated with anti-CD3 Ab (2.5 μg/ml) and APCs at 37°C with 5% CO_2_ for 5 days. APCs were T-cell-depleted syngeneic splenocytes as antigen-presenting cells expressing costimulatory molecules. In some groups, cells were also treated with either MR1 or CTLA4-Ig (2.5 μg/ml). The levels of IL-10 in the supernatant were measured using ELISA according to the manufacturer's instructions (Boster, China). The absorbance was read at 450 nm in a microplate spectrophotometer (Thermo Fisher Scientific, USA).

### Quantitative Real-Time PCR (qRT-PCR)

Total mRNA from skin grafts was isolated using Trizol reagents (Invitrogen, USA) and the mRNA was then transcribed to cDNA using a PrimeScript^TM^ RT reagent kit (TAKARA Bio Incorporation, Kusatsu, Japan) according to the instructions of the manufacturer. The cDNA was analyzed for the expression of cytokines using a Quantifast SYBR Green PCR kit (TAKARA Bio Incorporation) via an ABI 7,500 Fast RealTime PCR System (Thermo Fisher Scientific). The primer sequences were listed in the Table 1. The relative mRNA expression levels of cytokines were normalized against β-actin, and analysis was performed through a comparative 2^ΔΔ*CT*^ method. Values in control groups were set as 1.0, and all data were shown as relative mRNA expressions (fold changes).

**Table 1:** Primer sequences of target genes.

**Target genes**	**Primer sequences (5^**′**^ → 3^**′**^)**
IFN-**γ** (forward)	CACGGCACAGTCATTGAAAG
IFN-**γ** (reverse)	CATCCTTTTGCCAGTTCCTC
TNF-α (forward)	ACGGCATGGATCTCAAAGAC
TNF-α (reverse)	GTGGGTGAGGAGCACGTAGT
IL-6 (forward)	ACTTCCATCCAGTTGCCTTCTTGG
IL-6 (reverse)	TTAAGCCTCCGACTTGTGAAGTGG
β-Actin (forward)	TGTCCACCTTCCAGCAGATGT
β-Actin (reverse)	TGTCCACCTTCCAGCAGATGT

### Statistical Analysis

Comparisons of means were conducted using Student's *t*-test and one-way ANOVA. Data were presented as mean ± SD and analyzed through GraphPad Prism 6 (GraphPad Software, USA). The analysis of graft survival was performed using Kaplan–Meier method (log-rank test). A value of *P* < 0.05 was considered statistically significant.

## Results

### Administration of Ag-Specific CD8^+^CD122^+^PD-1^+^ Tregs Synergizes With Costimulatory Blockade of CD40/CD154, but Not B7/CD28, to Prolong Skin Allograft Survival

**Figure 1:**
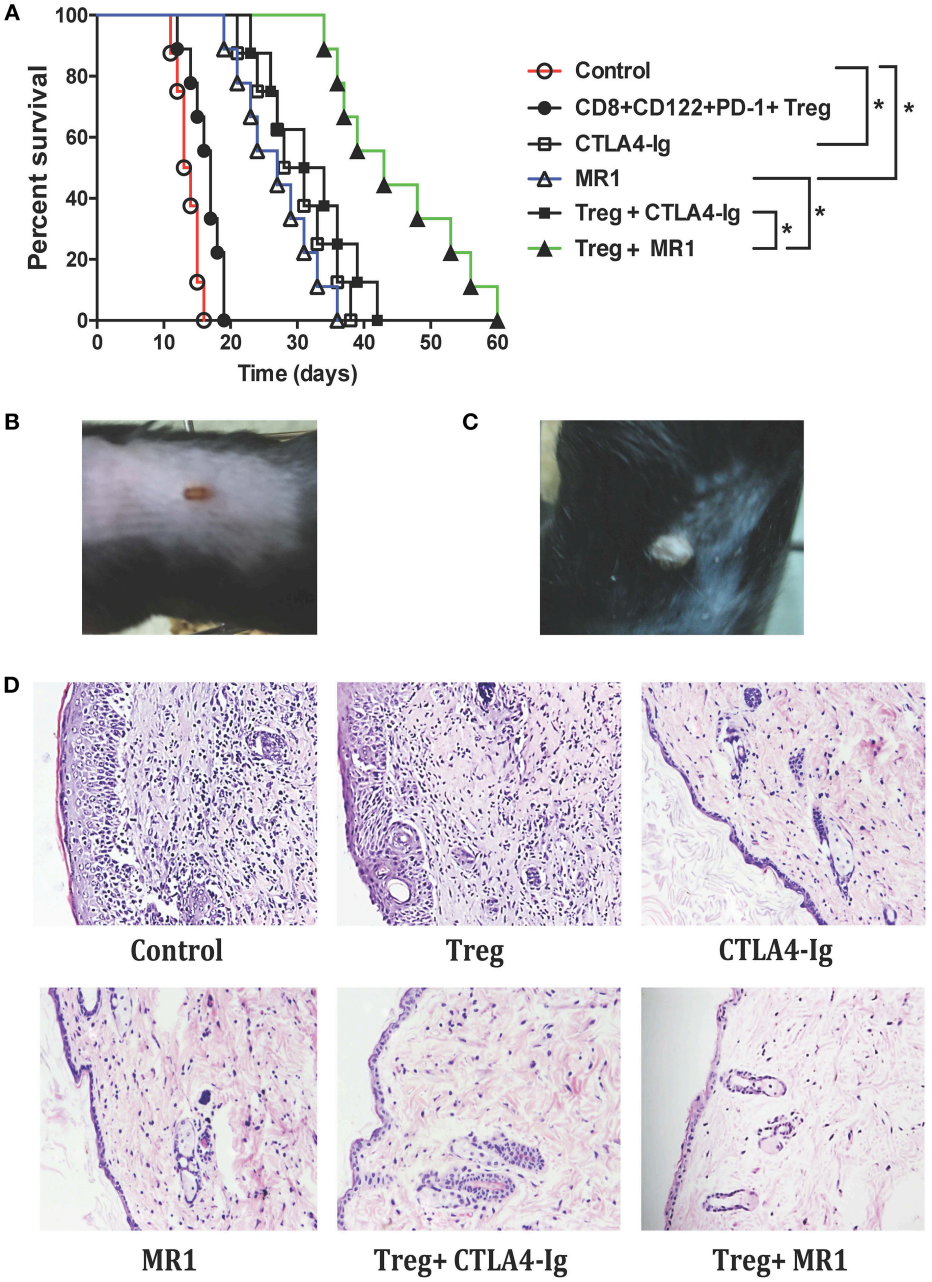
Administration of CD8^+^CD122^+^PD-1^+^ Tregs synergizes with costimulatory blockade of CD40/CD154, but not B7/CD28, to suppress allograft rejection. C57BL/6 mice that received syngeneic CD8^+^CD122^+^PD-1^+^ Tregs were transplanted with BALB/c skin and then treated with either MR1 (anti-CD154 Ab) or CTLA4-Ig, as described in the methods. Skin allograft rejection was observed (*n* = 8–9) **(A)**. A representative of rejected **(B)** and accepted **(C)** skin grafts was shown. H&E staining was also performed to evaluate cellular infiltration in skin grafts 2 weeks after skin transplantation. One set of images from three separate experiments is shown **(D)**. *P < 0.05.

To first generate and expand alloantigen-specific Tregs, FACS-sorted CD8^+^CD122^+^ T cells derived from C57BL/6 mice were cultured with irradiated and T-cell-depleted splenocytes from BALB/c mice in the absence or presence of recombinant IL-2 and/or IL-15 for 5 days. The percentages of PD-1^+^ cells within CD8^+^CD122^+^ population were determined via flow cytometric analysis. We also calculated the absolute numbers of CD8^+^CD122^+^PD-1^+^ Tregs. As shown in the supplementary data (Figure 1SA), addition of IL-2, but not IL-15, to the culture increased the percentages of PD-1^+^ cells within CD8^+^CD122^+^ population. Interestingly, either IL-2 or IL-15 alone augmented the absolute numbers of CD8^+^CD122^+^PD-1^+^ Tregs while IL-2 plus IL-15 further increased the Treg numbers compared to either cytokine alone (Figure 1SB), suggesting that both cytokines are needed to maximally induce and expand alloantigen-specific CD8^+^CD122^+^PD-1^+^ Tregs *in vitro*.

To boost the effects of CD8^+^CD122^+^PD-1^+^ Tregs on allograft survival in wild-type mice, C57BL/6 mice that received the syngeneic Tregs were transplanted with BALB/c skin and treated with either MR1 (anti-CD154 Ab) or CTLA4-Ig. As shown in Figure 1A, administration of CD8^+^CD122^+^PD-1^+^ Tregs alone did not significantly extend skin allograft survival in wild-type mice (MST = 17 vs. 14 days, *n* = 8–9, *P* > 0.05) while either CTLA4-Ig or MR1 treatment significantly prolonged the allograft survival (MST = 29 vs. 14 days or 27 vs. 14 days, *n* = 8–9, both *P* < 0.05). Interestingly, CD8^+^CD122^+^PD-1^+^ Tregs synergized with costimulatory blockade of CD40/CD154 (MST = 43 vs. 27, *n* = 8–9, *P* < 0.05), but not B7/CD28 (MST = 32 vs. 29, *n* = 8–9, *P* > 0.05), to prolong the allograft survival compared to the costimulatory blockade alone. As a control, isotype Ab did not alter skin allograft survival (data not shown). A representative of the rejected (Figure 1B) or accepted (Figure 1C) skin grafts was also shown. Furthermore, H&E staining exhibited a significant reduction in cellular infiltration in skin allografts after treatment with either CTLA4-Ig or MR1 while cellular infiltration was further diminished after simultaneous treatments with both CD8^+^CD122^+^PD-1^+^ Tregs and MR1 (Figure 1D).

### CD8^+^CD122^+^PD-1^+^ Tregs Cooperate With Costimulatory Blockade of CD40/CD154, but Not B7/CD28, to Inhibit T Cell Proliferation *in vitro*

**Figure 2:**
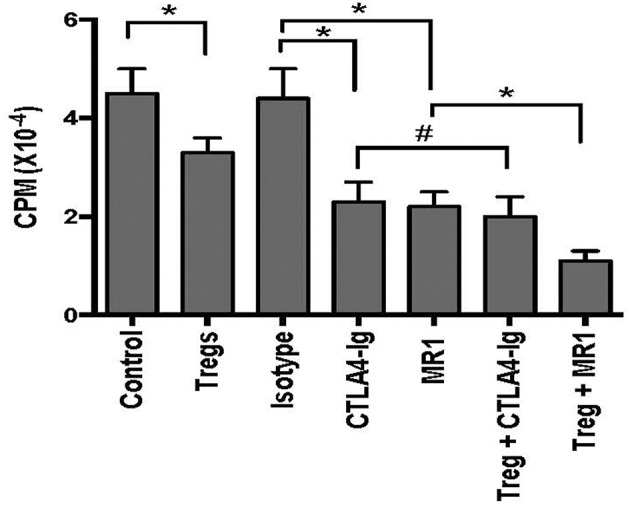
CD8^+^CD122^+^PD-1^+^ Treg suppression of T cell proliferation *in vitro*. MLRs were set up as described in the methods to measure CD8^+^CD122^+^PD-1^+^ Treg suppression of T cell proliferation *in vitro* in the presence of either MR1 or CTLA4-Ig. Cells were harvested and analyzed using a Scintillation counter 5 days after the culture. Data are presented as mean ± SD. One representative of three separate experiments is shown (*P < 0.05 and ^#^*P* > 0.05).

To determine if CD8^+^CD122^+^PD-1^+^ Tregs cooperate with the costimulatory blockade to enhance suppression of alloimmune responses *in vitro*, an MLR was set up to examine their joint effects on T cell proliferation *in vitro*. In the MLRs, nylonwood-enriched T cells were responders while CD8^+^CD122^+^PD-1^+^ Tregs were suppressors at a ratio of 1:2 (T suppressor vs. T responder). As shown in Figure 2, CD8^+^CD122^+^PD-1^+^ Tregs alone were capable of suppressing T cell proliferation to some extent while either CTLA4-Ig or MR1 dramatically inhibited T cell proliferation *in vitro*. However, the Tregs cooperated with costimulatory blockade of CD40/CD154 (MR1), but not B7/CD28 (CTLA4-Ig), to further suppress T cell proliferation compared to either costimulatory blockade alone (Figure 2). As a control, isotype Ab did not alter T cell proliferation *in vitro*.

### CD8^+^CD122^+^PD-1^+^ Tregs Cooperate With Costimulatory Blockade of CD40/CD154, but not B7/CD28, to Inhibit mRNA Expression of Proinflammatory Cytokines in Skin Allografts

**Figure 3:**
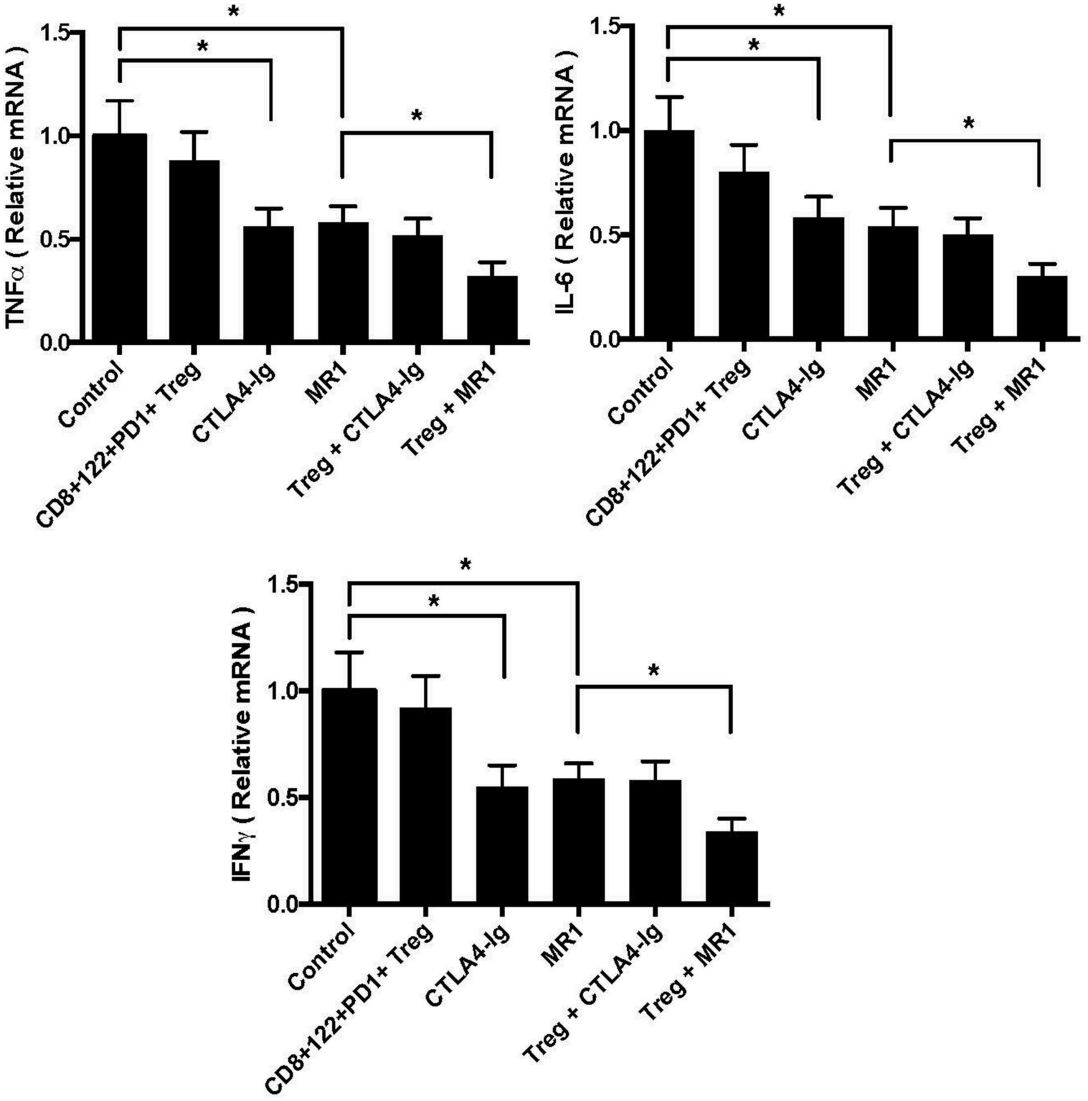
CD8^+^CD122^+^PD-1^+^ Tregs synergizes with costimulatory blockade of CD40/CD154, but not B7/CD28, to suppress mRNA expression of proinflammatory cytokines in skin allografts. mRNA expression of IFN-γ, TNF-α, and IL-6 in skin allografts was measured using RT-PCR assays 2 weeks after skin transplantation and treatment with CD8^+^CD122^+^PD-1^+^ Tregs, MR1, or CTLA4-Ig. Data are presented as mean ± SD. One representative of three separate experiments is shown (*P < 0.05).

To determine whether CD8^+^CD122^+^PD-1^+^ Tregs also cooperate with the costimulatory blockade in suppression of skin allograft inflammation, we measured mRNA expression of IFN-γ, TNF-α, and IL-6 in skin allografts through RT-PCR assays 2 weeks after skin transplantation. As shown in Figure 3, the mRNA levels of proinflammatory cytokines IFN-γ, TNF-α, and IL-6 in skin allografts were significantly downregulated after the treatment with either MR1 or CTLA4-Ig. Importantly, the Tregs cooperated with costimulatory blockade of CD40/CD154 (MR1), but not B7/CD28 (CTLA4-Ig), to further suppress the mRNA expression of these pro-inflammatory cytokines compared to either costimulatory blockade alone (Figure 3). These findings exhibited additive effects of CD8^+^CD122^+^PD-1^+^ Tregs and CD154 blockade in the context of alloimmune responsiveness.

### Costimulatory Blockade of B7/CD28, but Not CD40/CD154, Reduces the Numbers of Adoptively Transferred CD8^+^CD122^+^PD-1^+^ Tregs and Their Expression of IL-10

**Figure 4:**
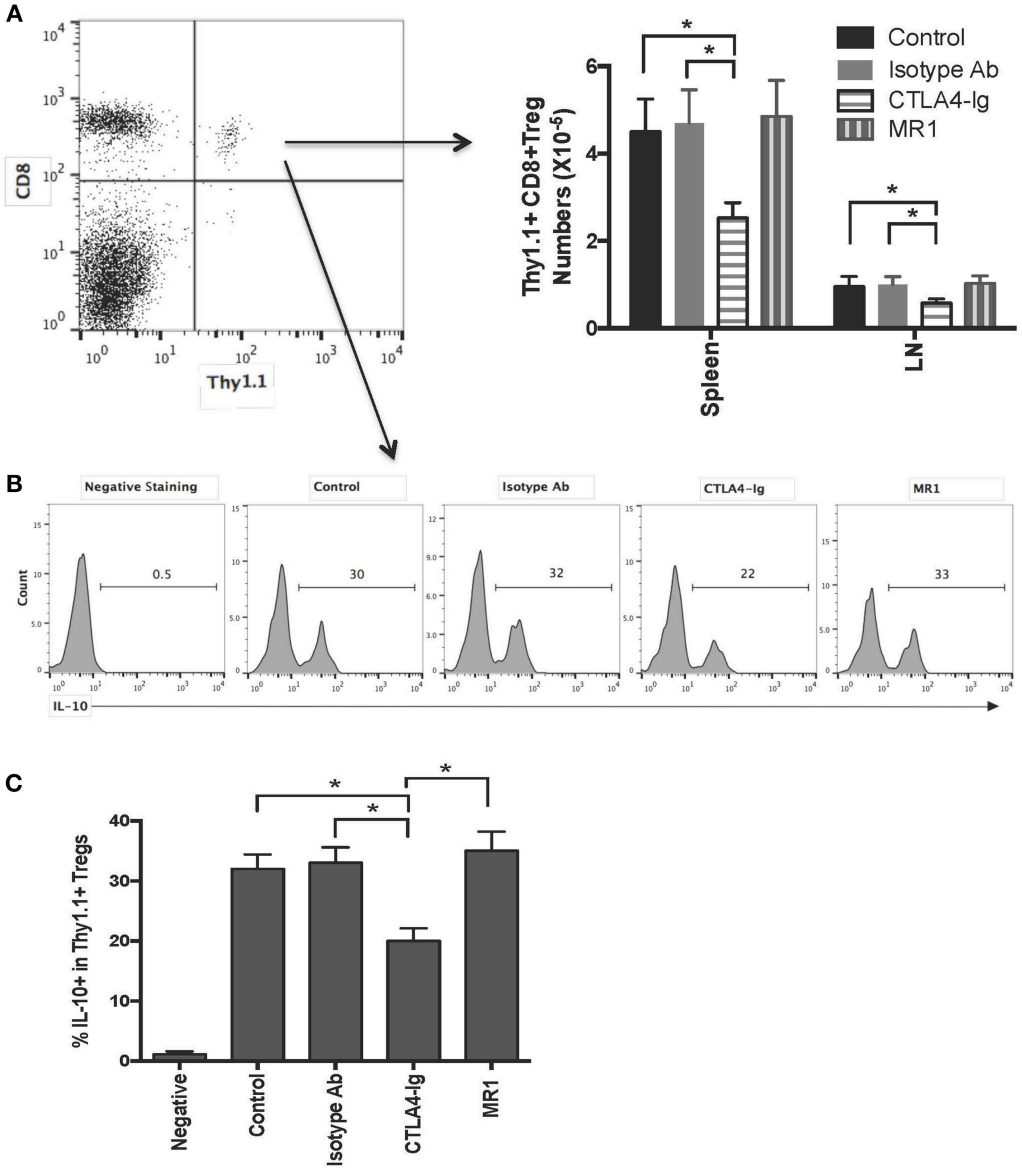
Costimulatory blockade of B7/CD28, but not CD40/CD154, reduces numbers of transferred CD8^+^CD122^+^PD-1^+^ Tregs and frequency of IL-10-expressing Tregs in recipient mice. C57BL/6 mice received CD8^+^CD122^+^PD-1^+^ Tregs derived from Thy1.1^+^ mice (C57BL/6 background). They were then transplanted with BALB/c skin and treated with either MR1 or CTLA4-Ig. Two weeks later, transferred CD8^+^Thy1.1^+^ Tregs were quantified via FACS analysis **(A)** or stained for intracellular expression of IL-10. Histograms showing percentages of IL-10^+^ cells were gated on transferred CD8^+^Thy1.1^+^ population with one representing set of FACS data **(B)**. Data are presented as mean ± SD from one representative of three separate experiments **(C)**. *P < 0.05.

To find out why there is an additive effect of CD8^+^CD122^+^PD-1^+^ Tregs and costimulatory blockade of CD40/CD154 but not B7/CD28, C57BL/6 mice received CD8^+^CD122^+^PD-1^+^ Tregs originally isolated from Thy1.1^+^ B6 mice and were transplanted with BALB/c skin. They were also treated with either MR1 or CTLA4-Ig. 2 weeks later, transferred Thy1.1^+^ Tregs in recipient mice were quantified via FACS analysis. As shown in Figure 4A, the numbers of transferred Thy1.1^+^CD8^+^ Tregs in both LNs and the spleen were much lower in recipient mice treated with CTLA4-Ig, but not MR1, than in those treated or untreated with the isotype Ab, suggesting that blocking B7/CD28, but not CD40/CD154, costimulation suppresses CD8^+^CD122^+^PD-1^+^ Treg expansion *in vivo*. On the other hand, intracellular staining also showed that blocking B7/CD28 (CTLA4-Ig), but not CD40/CD154 (MR1), costimulation reduced the frequency of transferred Thy1.1^+^CD8^+^ Tregs expressing IL-10 (Figures 4B,C).

### Costimulatory Blockade of B7/CD28, but Not CD40/CD154, Decreases IL-10 Production by CD8^+^CD122^+^PD-1^+^ Tregs *in vitro*

**Figure 5:**
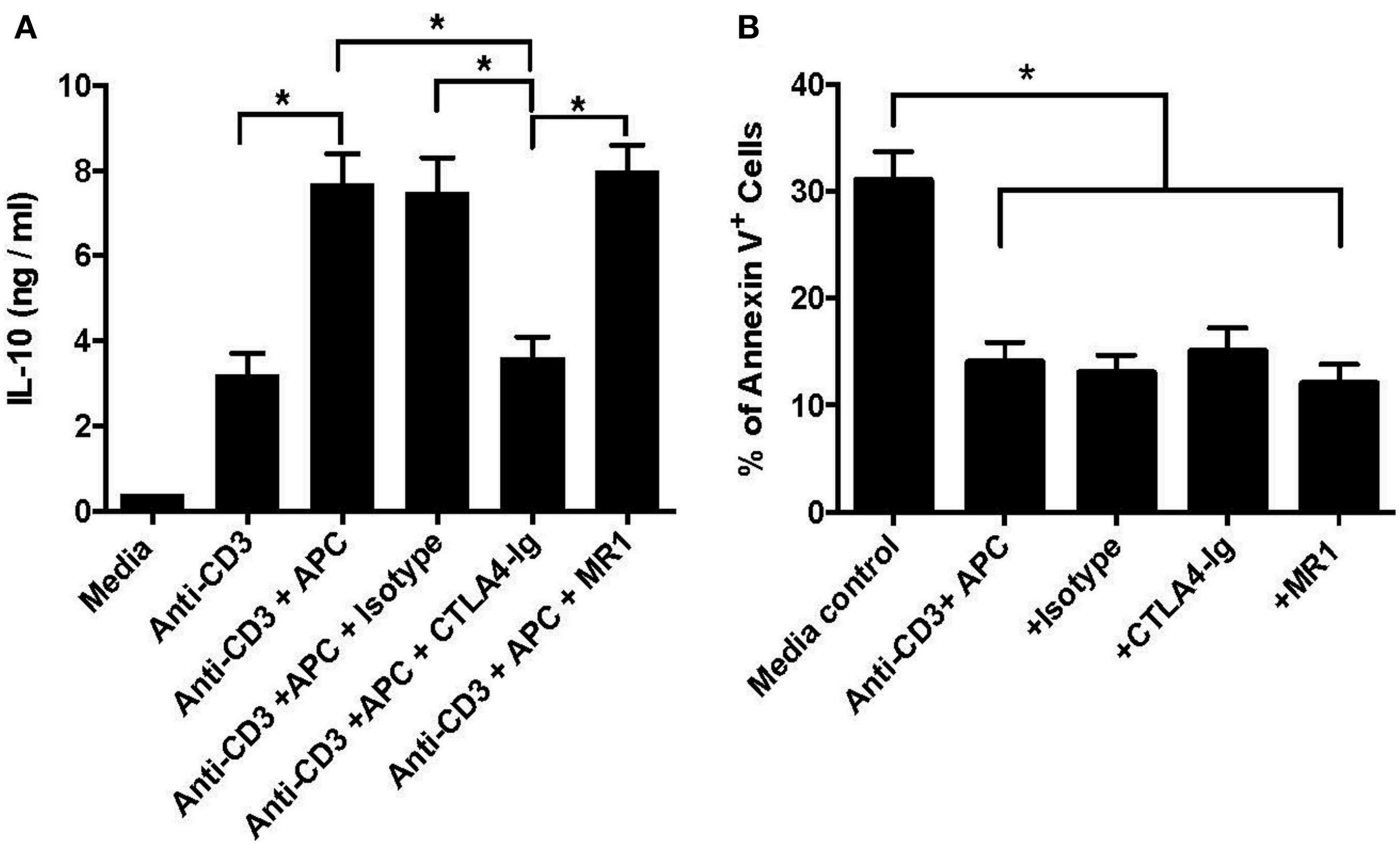
Costimulatory blockade of B7/CD28 decreases IL-10 production by CD8^+^CD122^+^PD-1^+^ Tregs *in vitro* but does alter their survival. CD8^+^CD122^+^PD-1^+^ Tregs were activated *in vitro* with anti-CD3 Ab in the presence of CD3-depleted splenocytes serving as syngeneic APCs. MR1 or CTLA4-Ig was also added to the culture in some groups. 5 days later, IL-10 production in the supernatant was measured via ELISA **(A)**. In other experiments, the similarly cultured cells were stained with annexin V and analyzed for their apoptosis **(B)**. Data are presented as mean ± SD. One representative of three separate experiments is shown (*P < 0.05).

Previous studies have shown that IL-10 is critical for the suppressive capacity of CD8^+^CD122^+^ Tregs. To determine whether the costimulatory blockade has an impact on IL-10 production by CD8^+^CD122^+^PD-1^+^ Tregs *in vitro*, they were activated with anti-CD3 Ab in the presence of CD3-depleted splenocytes serving as syngeneic APCs. Five days later, IL-10 production in the supernatant was measured via ELISA. As shown in Figure 5A, treatment with anti-CD3 Ab plus APCs further increased IL-10 levels compared to anti-CD3 Ab treatment alone. Importantly, blocking B7/CD28 (CTLA4-Ig), but not CD40/CD154 (MR1), costimulation significantly lowered IL-10 level in the supernatant compared with isotype Ab control, suggesting that B7/CD28, but not CD40/CD154, costimulatory pathway is also critical for the production of IL-10 by CD8^+^CD122^+^PD-1^+^ Tregs *in vitro*. On the other hand, the similarly cultured Tregs were stained with annexin V and analyzed for their death rates via FACS. As shown in Figure 5B, blocking either B7/CD28 or CD40/CD40L did not promote Treg apoptosis while Treg apoptotic rate in the media control group without activation was much higher than that in other groups with activation but without or with CTLA4-Ig/MR1. These data indicate that blocking either B7/CD28 or CD40/CD40L costimulation does not promote Treg apoptosis.

### Cooperation in Suppression of Allograft Rejection Between CD8^+^CD122^+^PD-1^+^ Tregs and Costimulatory Blockade of CD154 Is Largely Dependent on IL-10

**Figure 6:**
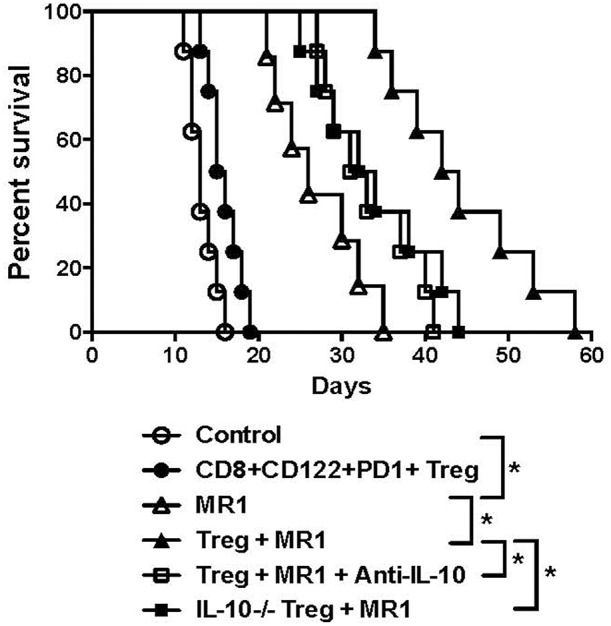
Synergy in suppression of allograft rejection between CD8^+^CD122^+^PD-1^+^ Tregs and costimulatory blockade of CD154 is largely dependent on IL-10. C57BL/6 mice that received IL-10-replete or IL-10-deficient CD8^+^CD122^+^PD-1^+^ Tregs were transplanted with BALB/c skin. They were also treated with MR1 (anti-CD154) while some received anti-IL-10 Ab to neutralize IL-10. Skin allograft rejection was observed (*n* = 7–8, *P < 0.05).

Since IL-10 plays an important role in CD8^+^CD122^+^ Treg-mediated suppression (5), we asked whether CD8^+^CD122^+^PD-1^+^ Tregs would cooperate with costimulatory blockade of CD40/CD154 to suppress allograft rejection in an IL-10-dependent manner. C57BL/6 mice received either IL-10-replete or IL-10-deficient CD8^+^CD122^+^PD-1^+^ Tregs and were transplanted with BALB/c skin. They were then treated with or without MR1. Some recipient mice were also treated with IL-10-neutralizing anti-IL-10 Ab following the Treg infusion. As shown in Figure 6, administration of the Tregs alone did not significantly alter skin allograft survival while MR1 indeed significantly extended the allograft survival. Moreover, MR1 plus Tregs further prolonged allograft survival compared to MR1 treatment alone. However, the additive effects of MR1 and the Tregs on suppression of skin allograft rejection was largely diminished when the Tregs lacked IL-10 or when recipient mice were treated with neutralizing anti-IL-10 Ab, indicating that cooperation between the Tregs and CD154 blockade is largely dependent on CD8^+^CD122^+^PD-1^+^ Treg-derived IL-10.

## Discussion

Tregs are essential to maintaining transplant tolerance (18–20). Although CD4^+^CD25^+^FoxP3^+^ Tregs are a major subset of Tregs (21–25), mounting evidence has shown that CD8^+^CD122^+^ T cells also are Tregs that suppress T cell responses (4–9) and autoimmunity (10, 11). Deletion of the transcription factor Foxp3 (24, 25) has diminished the development and function of conventional CD4^+^CD25^+^ Tregs. We have demonstrated that CD8^+^CD122^+^ T cells are Tregs (16, 26, 27) that are even more effective in suppression of allograft rejection than their CD4^+^CD25^+^ counterparts (15). Further, PD-1^+^ component within CD8^+^CD122^+^ population is largely responsible for CD8^+^CD122^+^ Treg-mediated suppression (16). However, a common problem with Treg therapies is that infusion of Tregs alone does not achieve satisfactory outcome in immune-competent wild-type animals. Therefore, it's important to enhance their potency of immunosuppression in normal animals or humans by combining other measures, including costimulatory blockade. Here, we found that administration of CD8^+^CD122^+^PD-1^+^ Tregs synergized with costimulatory blockade of CD40/CD154, but not B7/CD28, to prolong allograft survival in wild-type mice. Blocking CD40/CD154, but not B7/CD28, also cooperated with the Tregs to inhibit T cell proliferation *in vitro*. Thus, this finding may be implicated in clinical transplantation.

Based on our present study, it remains not totally clear why CD8^+^CD122^+^PD-1^+^ Tregs cooperated with costimulatory blockade of CD40/CD154, but not B7/CD28, in suppression of allograft rejection and T-cell proliferation. We found that costimulatory blockade of B7/CD28, but not CD40/CD154, indeed reduced the numbers of transferred Thy1.1^+^ Tregs in recipient mice, suggesting that B7/CD28, but not CD40/CD154, costimulation is important for homeostatic proliferation or expansion of CD8^+^CD122^+^PD-1^+^ Tregs. On the other hand, we discovered that the costimulatory blockade of B7/CD28, but not CD40/CD154, decreased IL-10 production by CD8^+^CD122^+^PD-1^+^ Tregs in the presence of CD3 signaling and APCs *in vitro* and also reduced IL-10 expression in transferred CD8^+^CD122^+^PD-1^+^ Tregs *in vivo*, indicating that B7/CD28 costimulation is required for the Tregs to produce IL-10. These results may explain why there is no cooperation in their suppression of alloimmunity between the Tregs and B7/CD28 blockade given that IL-10 produced by the Tregs plays an important role in CD8^+^CD122^+^PD1^+^ Treg-mediated suppression and that B7/CD28 blockade inhibits their production of IL-10. On the other hand, we found that B7/CD28 blockade did not accelerate the Treg apoptosis *in vitro*. Therefore, B7/CD28 costimulation is required for Treg expansion and function, which may explain why B7/CD28 blockade and the Treg therapies should not be combined for suppression of allograft rejection. It's also unclear why transfer of the Tregs alone does not satisfyingly extend allograft survival in immune-competent mice, but became highly effective under the cover of CD154 blockade. Perhaps, the adoptive transfer of the Tregs is insufficient to inhibit vigorously acute alloimmune responses in normal animals whereas the effects of the Tregs may emerge only after acute allograft rejection is suppressed by CD154 costimulatory blockade.

Programmed death-1 (PD-1) is a co-inhibitory molecule that is essential for the maintenance of immune tolerance and suppression of autoimmune responses (28–34). Previous studies have also shown that PD-1 molecule on CD8^+^ T cells is mainly responsible for their exhaustion during chronic viral infection while blocking PD-1 has restored the function of exhausted CD8^+^ T cells (35–38). Based on results in our present study, it is possible that some of the exhausted CD8^+^ T cells expressing PD-1 may be Tregs that are likely responsible for depressed immunity against chronic viral infection and the persistence of the viral infection.

It is well accepted that antigen-specific Tregs are superior to polyclonal Tregs in their suppression of alloimmunity and autoimmunity (26, 39, 40) because the former can respond vigorously and also rapidly and specifically migrate to a target organ. Antigen-specific Tregs are also expected to cause less side effects resulting from systemic immunosuppression than polyclonal Tregs (41). In the present study, we utilized CD8^+^CD122^+^PD-1^+^ Tregs that were expanded *in vitro* in the presence of donor splenocytes and recombinant IL-2/IL-15. Donor-antigen-specific expansion likely has ensured their allospecificity and efficacy in suppression of alloimmune responses. On the other hand, addition of IL-2 to the cell culture increased the frequency of PD-1^+^ components while IL-15 further enhanced the expansion of the total Treg population, indicating that they are memory-like CD8^+^CD122^+^ Tregs. It remains to be confirmed whether these antigen-expanded Tregs are actually more potent in suppression of allograft rejection than their polyclonal counterparts.

In conclusion, there is clearly a cooperation between infusion of CD8^+^CD122^+^PD-1^+^ Tregs and costimulatory blockade of CD40/CD154, but not B7/CD28, in suppression of allograft rejection in immune-competent wild-type mice. The Tregs and CD154 blockade also exhibit an additive effect on suppression of T cell proliferation *in vitro* and pro-inflammatory cytokine expression in skin allografts. Furthermore, costimulatory blockade of B7/CD28, but not CD40/CD154, has a negative impact on adoptively transferred CD8^+^CD122^+^PD-1^+^ Treg expansion *in vivo* and their production of IL-10. These results may lay the groundwork for a clinical trial for inhibition of human allograft rejection by combined therapies with CD8^+^CD122^+^PD-1^+^ Tregs and CD154 costimulatory blockade.

## Ethics Statement

This study was carried out in accordance with the recommendations of national guidelines of China, and the protocol was approved by the Institutional Animal Ethical Committee of Guangdong Provincial Academy of Chinese Medical Sciences.

## Author Contributions

HL and FQ performed experiments and wrote the partial manuscript. YW, QZ, and CL performed some experiments. YC, C-LL, and QFZ analyzed data. LH and ZD wrote or edited the manuscript.

### Conflict of Interest Statement

The authors declare that the research was conducted in the absence of any commercial or financial relationships that could be construed as a potential conflict of interest.

## References

[1] Morris PJ. Transplantation–a medical miracle of the 20th century. N Engl J Med. (2004) 351:2678–80. 10.1056/NEJMp048256 15616201

[2] Lechler RI Sykes M Thomson AW Turka LA Organ transplantation–how much of the promise has been realized? Nat Med. (2005) 11:605–13. 10.1038/nm1251 15937473

[3] Halloran PF. Immunosuppressive drugs for kidney transplantation. N Engl J Med. (2004) 351:2715–29. 10.1056/NEJMra033540 15616206

[4] Rifa'i M Kawamoto Y Nakashima I Suzuki H. Essential roles of CD8+CD122+ regulatory T cells in the maintenance of T cell homeostasis. J Exp Med. (2004) 200:1123–34. 10.1084/jem.20040395 15520244 PMC2211869

[5] Endharti AT Rifa IM Shi Z Fukuoka Y Nakahara Y Kawamoto Y. Cutting edge: CD8+CD122+ regulatory T cells produce IL-10 to suppress IFN-gamma production and proliferation of CD8+ T cells. J Immunol. (2005) 175:7093–7. 10.4049/jimmunol.175.11.7093 16301610

[6] Chen X Priatel JJ Chow MT Teh HS. Preferential development of CD4 and CD8 T regulatory cells in RasGRP1-deficient mice. J Immunol. (2008) 180:5973–82. 10.4049/jimmunol.180.9.5973 18424717

[7] Shi Z Rifa'i M Lee YH Shiku H Isobe K Suzuki H. Importance of CD80/CD86-CD28 interactions in the recognition of target cells by CD8+CD122+ regulatory T cells. Immunology (2008) 124:121–8. 10.1111/j.1365-2567.2007.02747.x 18205792 PMC2434386

[8] Molloy MJ Zhang W Usherwood EJ. Suppressive CD8+ T cells arise in the absence of CD4 help and compromise control of persistent virus. J Immunol. (2011) 186:6218–26. 10.4049/jimmunol.1003812 21531895 PMC3854932

[9] Endharti AT Okuno Y Shi Z Misawa N Toyokuni S Ito M. CD8+CD122+ regulatory T cells (Tregs) and CD4+ Tregs cooperatively prevent and cure CD4+ cell-induced colitis. J Immunol. (2011) 186:41–52. 10.4049/jimmunol.1000800 21098236

[10] Kim HJ Wang X Radfar S Sproule TJ Roopenian DC Cantor H. CD8+ T regulatory cells express the Ly49 Class I MHC receptor and are defective in autoimmune prone B6-Yaa mice. Proc Nat Acad Sci USA. (2011) 108:2010–5. 10.1073/pnas.1018974108 21233417 PMC3033298

[11] Mangalam AK Luckey D Giri S Smart M Pease LR Rodriguez M. Two discreet subsets of CD8 T cells modulate PLP(91-110) induced experimental autoimmune encephalomyelitis in HLA-DR3 transgenic mice. J Autoimmun. (2012) 38:344–53. 10.1016/j.jaut.2012.02.004 22459490 PMC3581307

[12] Lee YH Ishida Y Rifa'i M Shi Z Isobe K Suzuki H. Essential role of CD8+CD122+ regulatory T cells in the recovery from experimental autoimmune encephalomyelitis. J Immunol. (2008) 180:825–32. 10.4049/jimmunol.180.2.825 18178821

[13] Seifert HA Benedek G Nguyen H Kent G Vandenbark AA Offner H. Estrogen protects both sexes against EAE by promoting common regulatory cell subtypes independent of endogenous estrogen. Metab Brain Dis. (2017) 32:1747–54. 10.1007/s11011-017-0063-8 28689297 PMC5650507

[14] Saitoh O Abiru N Nakahara M Nagayama Y. CD8+CD122+ T cells, a newly identified regulatory T subset, negatively regulate Graves' hyperthyroidism in a murine model. Endocrinology (2007) 148:6040–6. 10.1210/en.2007-0300 17823258

[15] Dai Z Zhang S Xie Q Wu S Su J Li S. Natural CD8+CD122+ T cells are more potent in suppression of allograft rejection than CD4+CD25+ regulatory T cells. Am J Transplant. (2014) 14:39–48. 10.1111/ajt.12515 24219162

[16] Dai H Wan N Zhang S Moore Y Wan F Dai Z. Cutting edge: programmed death-1 defines CD8+CD122+ T cells as regulatory versus memory T cells. J Immunol. (2010) 185:803–7. 10.4049/jimmunol.1000661 20548035

[17] Dai Z Li Q Wang Y Gao G Diggs LS Tellides G. CD4+CD25+ regulatory T cells suppress allograft rejection mediated by memory CD8+ T cells via a CD30-dependent mechanism. J Clin Invest. (2004) 113:310–7. 10.1172/JCI19727 14722622 PMC311434

[18] Edozie FC Nova-Lamperti EA Povoleri GA Scotta C John S Lombardi G. Regulatory T-cell therapy in the induction of transplant tolerance: the issue of subpopulations. Transplantation (2014) 98:370–9. 10.1097/TP.0000000000000243 24933458

[19] Safinia N Scotta C Vaikunthanathan T Lechler RI Lombardi G. Regulatory T cells: serious contenders in the promise for immunological tolerance in transplantation. Front Immunol. (2015) 6:438. 10.3389/fimmu.2015.00438 26379673 PMC4553385

[20] Tang Q Vincenti F. Transplant trials with tregs: perils and promises. J Clin Invest. (2017) 127:2505–12. 10.1172/JCI90598 28665300 PMC5490750

[21] Asano M Toda M Sakaguchi N Sakaguchi S. Autoimmune disease as a consequence of developmental abnormality of a T cell subpopulation. J Exp Med. (1996) 184:387–96. 10.1084/jem.184.2.387 8760792 PMC2192701

[22] Shevach EM McHugh RS Thornton AM Piccirillo C Natarajan K Margulies DH. Control of autoimmunity by regulatory T cells. Adv Exp Med Biol. (2001) 490:21–32. 10.1007/978-1-4615-1243-1_3 11505971

[23] Qin S Cobbold SP Pope H Elliott J Kioussis D Davies J. “Infectious” transplantation tolerance. Science (1993) 259:974–7. 10.1126/science.8094901 8094901

[24] Hori S Nomura T Sakaguchi S. Control of regulatory T cell development by the transcription factor Foxp3. Science (2003) 299:1057–61. 10.1126/science.1079490 12522256

[25] Fontenot JD Gavin MA Rudensky AY. Foxp3 programs the development and function of CD4+CD25+ regulatory T cells. Nat Immunol. (2003) 4:330–6. 10.1038/ni904 12612578

[26] Liu J Chen D Nie GD Dai Z. CD8(+)CD122(+) T-Cells: a newly emerging regulator with central memory cell phenotypes. Front Immunol. (2015) 6:494. 10.3389/fimmu.2015.00494 26539191 PMC4610204

[27] Qiu F Liu H Liang CL Nie GD Dai Z. A new immunosuppressive molecule emodin induces both CD4(+)FoxP3(+) and CD8(+)CD122(+) regulatory t cells and suppresses murine allograft rejection. Front Immunol. (2017) 8:1519. 10.3389/fimmu.2017.01519 29167674 PMC5682309

[28] Nishimura H Okazaki T Tanaka Y Nakatani K Hara M Matsumori A. Autoimmune dilated cardiomyopathy in PD-1 receptor-deficient mice. Science (2001) 291:319–22. 10.1126/science.291.5502.319 11209085

[29] Ansari MJI Salama AD Chitnis T Smith RN Yagita H Akiba H. The programmed death-1 (PD-1) pathway regulates autoimmune diabetes in nonobese diabetic (NOD) mice. J Exp Med. (2003) 198:63–9. 10.1084/jem.20022125 12847137 PMC2196083

[30] Keir ME Liang SC Guleria I Latchman YE Qipo A Albacker LA. Tissue expression of PD-L1 mediates peripheral T cell tolerance. J Exp Med. (2006) 203:883–95. 10.1084/jem.20051776 16606670 PMC2118286

[31] Francisco LM Salinas VH Brown KE Vanguri VK Freeman GJ Kuchroo VK. PD-L1 regulates the development, maintenance, and function of induced regulatory T cells. J Exp Med. (2009) 206:3015–29. 10.1084/jem.20090847 20008522 PMC2806460

[32] Gao W Demirci G Strom TB Li XC Stimulating PD-1-negative signals concurrent with blocking CD154 costimulation induces long-term islet allograft survival. Transplantation (2003) 76:994–9. 10.1097/01.TP.0000085010.39567.FB 14508368

[33] Koehn BH Ford ML Ferrer IR Borom K Gangappa S Kirk AD. PD-1-dependent mechanisms maintain peripheral tolerance of donor-reactive CD8+ T cells to transplanted tissue. J Immunol. (2008) 181:5313–22. 10.4049/jimmunol.181.8.5313 18832687 PMC2572818

[34] Haspot F Fehr T Gibbons C Zhao G Hogan T Honjo T Peripheral deletional tolerance of alloreactive CD8 but not CD4 T cells is dependent on the PD-1/PD-L1 pathway. Blood (2008) 112:2149–55. 10.1182/blood-2007-12-127449 18577709 PMC2518911

[35] Velu V Titanji K Zhu B Husain S Pladevega A Lai L. Enhancing SIV-specific immunity *in vivo* by PD-1 blockade. Nature (2009) 458:206–10. 10.1038/nature07662 19078956 PMC2753387

[36] Blackburn SD Shin H Haining WN Zou T Workman CJ Polley A. Coregulation of CD8+ T cell exhaustion by multiple inhibitory receptors during chronic viral infection. Nat Immunol. (2009) 10:29–37. 10.1038/ni.1679 19043418 PMC2605166

[37] Barber DL Wherry EJ Masopust D Zhu B Allison JP Sharpe AH. Restoring function in exhausted CD8 T cells during chronic viral infection. Nature (2006) 439:682–7. 10.1038/nature04444 16382236

[38] Petrovas C Casazza JP Brenchley JM Price DA Gostick E Adams WC. PD-1 is a regulator of virus-specific CD8+ T cell survival in HIV infection. J Exp Med. (2006) 203:2281–92. 10.1084/jem.20061496 16954372 PMC2118095

[39] Pothoven KL Kheradmand T Yang Q Houlihan JL Zhang H Degutes M. Rapamycin-conditioned donor dendritic cells differentiate CD4CD25Foxp3 T cells *in vitro* with TGF-beta1 for islet transplantation. Am J Transplant. (2010) 10:1774–84. 10.1111/j.1600-6143.2010.03199.x 20626386 PMC3995630

[40] Zheng J Liu Y Qin G Chan PL Mao H Lam KT. Efficient induction and expansion of human alloantigen-specific CD8 regulatory T cells from naive precursors by CD40-activated B cells. J Immunol. (2009) 183:3742–50. 10.4049/jimmunol.0901329 19684082

[41] Zhang Q Lu W Liang CL Chen Y Liu H Qiu F. Chimeric antigen receptor (CAR) treg: a promising approach to inducing immunological tolerance. Front Immunol. (2018) 9:2359. 10.3389/fimmu.2018.02359 30369931 PMC6194362

