# Supplementary material for: CD8+CD122+PD-1+ Tregs Synergize With Costimulatory Blockade of CD40/CD154, but Not B7/CD28, to Prolong Murine Allograft Survival
Source: Front Immunol. 2019 Feb 26;10:306. doi: 10.3389/fimmu.2019.00306 (PMC6399415; doi:10.3389/fimmu.2019.00306)
Supplement: Supplementary file 1 [file Data_Sheet_1.PDF]

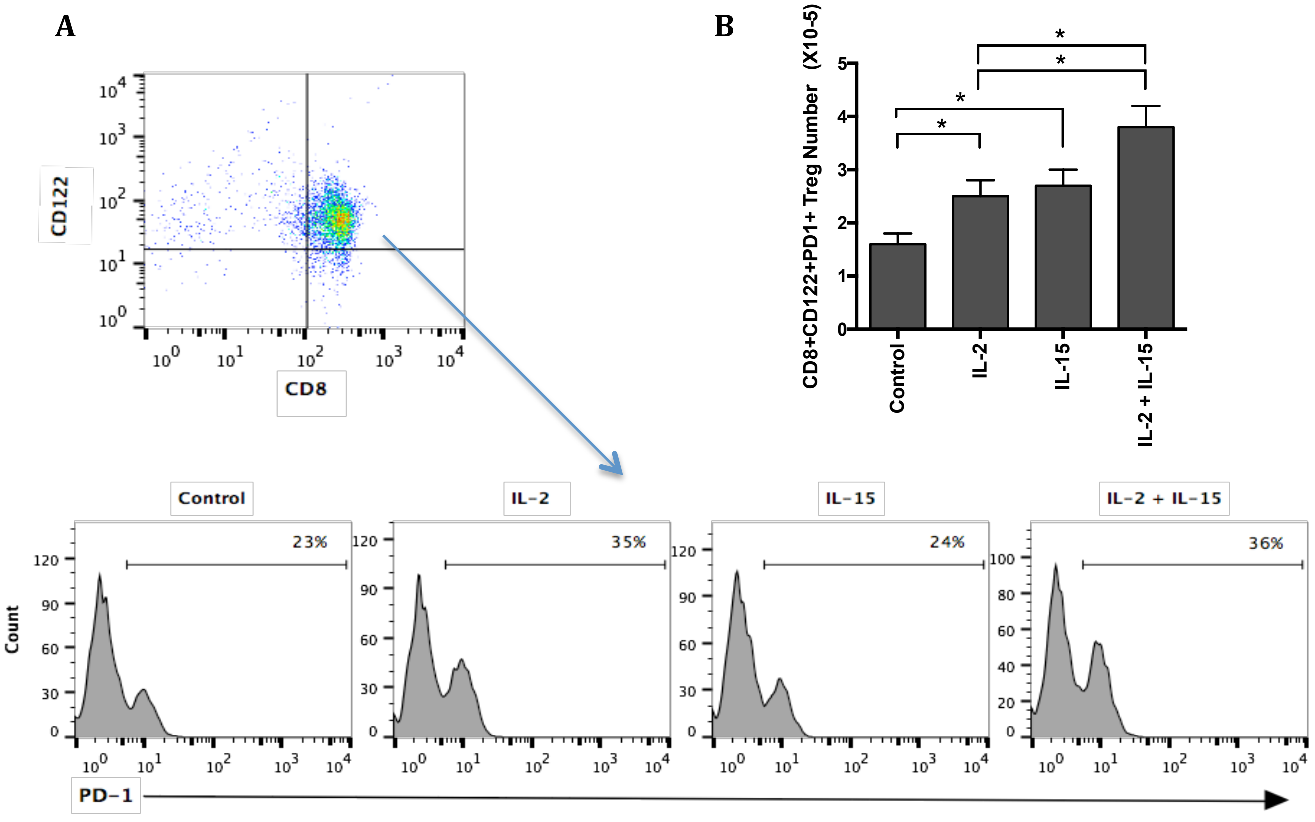

Figure 1S. In vitro expansion and induction of CD8+CD122+PD-1+ Tregs
FACS-sorted CD8+CD122+ cells derived from C57BL/6 mice were cultured with irradiated and T-cell-depleted splenocytes from BALB/c mice in the absence or presence of recombinant IL-2 and/or IL-15 for five days. (**A**) The percentages of PD-1+ cells within CD8+CD122+ population were determined via flow cytometric analysis after originally gating on live lymphocyte population. (**B**) Also calculated were the absolute numbers per well of CD8+CD122+PD-1+ Tregs five days after the culture. One representative of three separate analyses is shown (*$P<0.05$ vs. Control). Data indicate that IL-2 increases the number of the triple-positive Tregs mainly by augmenting PD-1+ cell frequency while IL-15 expands the total of CD8+CD122+ T cells, including the PD-1+ component.
